# Epithelial to mesenchymal transition in tumor cells as consequence of phenotypic instability

**DOI:** 10.3389/fcell.2014.00071

**Published:** 2014-12-12

**Authors:** Antonio García de Herreros

**Affiliations:** Programa de Recerca en Càncer, Departament de Ciències Experimentals i de la Salut, Institut Hospital del Mar d'investigacions Mèdiques, Universitat Pompeu FabraBarcelona, Spain

**Keywords:** EMT, Snail1, E-cadherin, self-amplification, tumor instability

## Abstract

During the last years many articles have reported epithelial-to-mesenchymal transitions (EMT) induced by a myriad of gene products either when added to the cell medium or when transfected. Molecularly the EMT is characterized by the up-regulation of transcriptional factors (EMT-TFs) repressing the epithelial gene E-cadherin, a protein essential for the maintenance of the epithelial phenotype. These EMT-TFs are subjected to a complex regulation involving binary self-stimulatory loops, allowing the possibility of the amplification of input signals. The capability of EMT-TFs to promote an EMT is controlled by E-cadherin that limits the transcription of mesenchymal genes. We discuss here the differences between normal and tumor epithelial cells; in the latter a partial inactivation of E-cadherin function enables extracellular signals to be amplified and induce an EMT. This tumor cell phenotypic instability is exacerbated in cell culture conditions. Therefore, it is likely that many of the gene products reported to control this transition act only in very specific cell tumor cell lines; thus, in cells with an unstable phenotype due to pre-existing alterations in E-cadherin safeguard mechanism.

## Transcriptional factors control EMT

Epithelial to Mesenchymal transition (EMT) was initially described by Elizabeth Hay decades ago (Hay, 1968); in the 80's and 90's several groups reported the acquisition of a fibroblastic, scattered or undifferentiated phenotype after prolonged treatments of epithelial cells with cytokines such as TGF-β. EMT is characterized by an extensive down-modulation of the key epithelial gene E-cadherin and the induction of mesenchymal markers, such as Fibronectin or Vimentin (Nieto, 2011). However, it was at the beginning of 2000 when EMT started to be widely studied following the description of transcriptional repressors that directly act on E-cadherin gene, inhibiting its expression and inducing the acquisition of a mesenchymal phenotype by epithelial cells (Batlle et al., 2000; Cano et al., 2000; Grooteclaes and Frisch, 2000; Comijn et al., 2001; Hajra et al., 2002; Yang et al., 2004). Among these transcriptional factors, the Snail family, particularly Snail1, has received more attention although the Zeb and Twist families have also been extensively studied. All these factors will be referred below as EMT-related transcription factors (EMT-TFs).

Transfection of Snail1, Zeb1 or Twist1 induces an EMT in most cell lines (Batlle et al., 2000; Cano et al., 2000; Grooteclaes and Frisch, 2000; Comijn et al., 2001; Hajra et al., 2002; Yang et al., 2004). When compared, Zeb1 is more potent as E-cadherin repressor whereas Twist induces better mesenchymal genes; Snail1 seems to gather both properties (Garcia de Herreros and Baulida, 2012). Snail1 ectopic expression induces Zeb1 expression; however, Zeb1 does not alter Snail1 levels which suggests that Zeb1 (Nieto, 2011) is downstream Snail1 in the signaling pathway controlling EMT (Garcia de Herreros and Baulida, 2012). Twist and Snail1 seem to reside in parallel branches since only very occasionally they activate each other (at least transcriptionally), although both cooperatively induce Zeb1 (Dave et al., 2011). The complementary role of both factors is also demonstrated by developmental studies indicating that during gastrulation Twist hypomorphic mutants are rescued by Snail1 overexpression (Wong et al., 2014).

Kinetically Snail1 is the first factor to be up-regulated during cytokine-driven g EMT (Peinado et al., 2007). For instance, in the broadly used system of mammary murine NMuMG cells exposed to TGF-β, Snail1 up-regulation is detected as early as 30 min after TGF-β addition whereas Zeb1 or other mesenchymal markers require 4–6 h and Twist1 is not consistently increased (Dave et al., 2011). This activation of Snail1 protein is transient whereas that of Zeb1 is not. Due to these results Snail1 has been considered as the transcriptional factor triggering EMT, whereas Zeb1 would be responsible for the consolidation of the mesenchymal phenotype by repressing E-cadherin and other epithelial genes (Peinado et al., 2007; Garcia de Herreros and Baulida, 2012).

The EMT-TFs present common features. For instance all Snail1/2, Zeb1/2 and Twist are very unstable proteins with half-lives around 30 min (Diaz et al., in press). Different ubiquitin ligases have been characterized acting specifically on one, two, or even three of these factors (Diaz et al., in press); therefore, down-regulation of these ubiquitin ligases is capable to trigger EMT (Viñas-Castells et al., 2014; Zheng et al., 2014).

## Signaling pathways controlling EMT show positive and negative feed-back loops

EMT-TFs gene expression is dependent on the activity of several common and ubiquitous transcriptional factors, such as NF-κB, β-catenin/Tcf-4 and Ets-2 (Garcia de Herreros and Baulida, 2012; De Craene and Berx, 2013). Since activation of these factors can be achieved by several extracellular cues, it should be expected that EMT is induced by these signals although this is not the case; for instance Snail1 transcription can be up-regulated by factors, such as TNFα or Wnt3a, that normally do not promote an EMT. It is possible that the extent, or more likely the continuity, of the stimulation might be relevant and a persistent up-regulation, for instance in β-catenin/Tcf-4 transcriptional activity, might promote an EMT. Alternatively, this transition might also require the convergence of signals acting on two of these transcriptional complexes, or the activation of not only the transcription but also the stabilization of key genes such as Snail1. This can also apply to some other levels of control since the mRNA stability of many EMT-TFs is also controlled by miRNAs.

**Figure 1:**
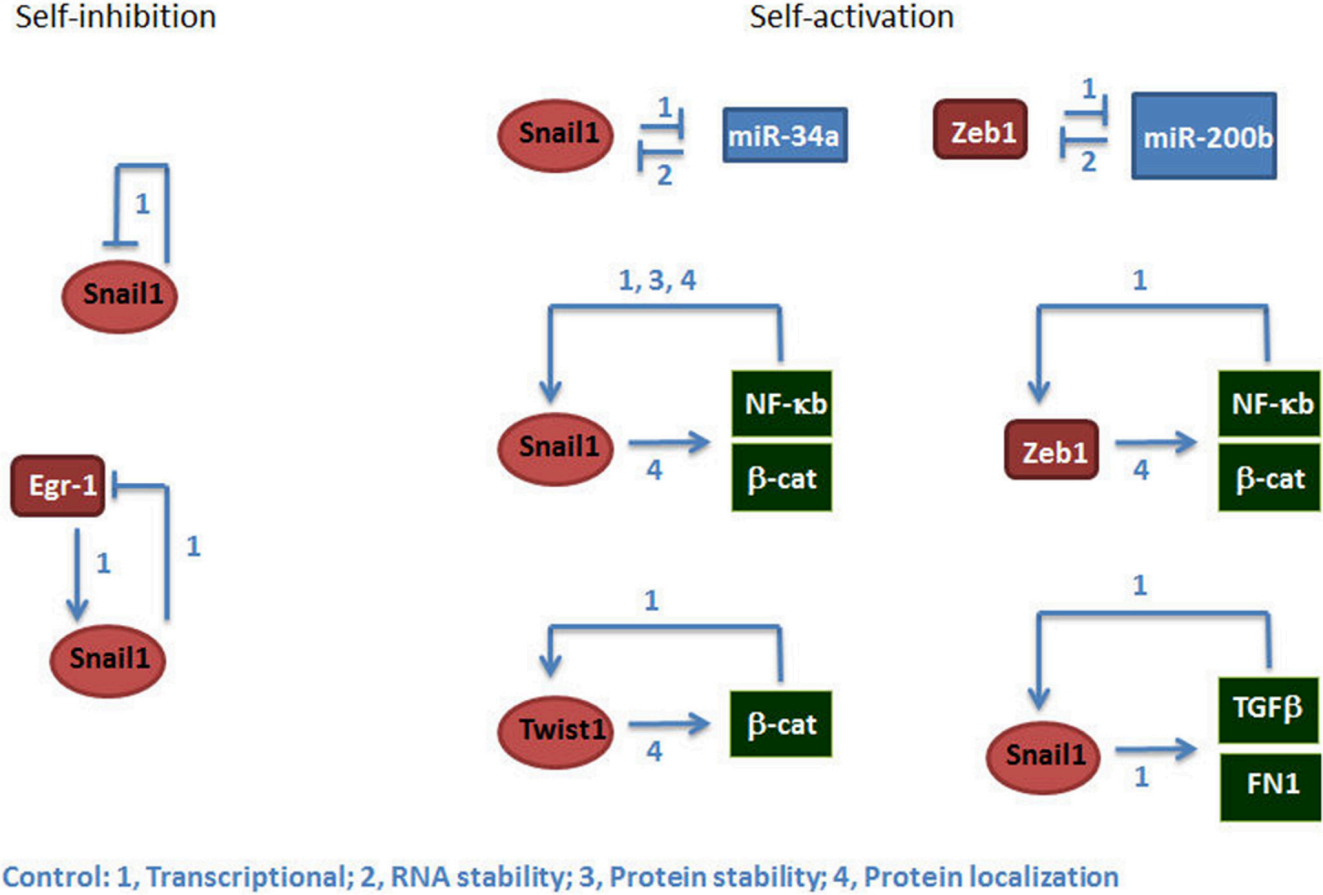
**EMT-TFs are controlled by self-inhibition and self-activation**. The figure shows examples of mechanisms of self-inhibition **(left)** or activation **(right)** controlling the expression of Snail1, Twist1, and Zeb1. The number indicates the type of control. Besides the references indicated in the text, the mechanisms shown in this figure are documented in Howe et al. (2003), Barberà et al. (2004), Bachelder et al. (2005), Yook et al. (2006), Julien et al. (2007), Stemmer et al. (2008), Kudo-Saito et al. (2009), Wu et al. (2009), Sánchez-Tillo et al. (2011).

Moreover, the study of the transduction pathways controlling the expression of EMT-TFs has revealed that these pathways are not linear but display numerous points of self-control either positive or negative. It has been demonstrated the existence of binary circuits composed by two genes that negatively regulate each other; for instance in the systems miR-34a/Snail1 and miR-200/Zeb1, the EMT-TF inhibits the miRNA acting on the same TF (Bracken et al., 2008; Burk et al., 2008; Kim et al., 2011; Siemens et al., 2011) (Figure 1). This creates a positive feed-back loop since increases in Snail1, for instance, inhibits its repressor miR-34a and further stimulates Snail1 mRNA stability. Other examples are provided by the action of the transcriptional complexes mentioned above; stimulation of the canonical Wnt pathway induces the repression of E-cadherin through the stimulation of β–catenin/TCF-4 dependent-EMT-TFs (such as Zeb1), releasing more β-catenin and enhancing the activity of this transcriptional complex, therefore amplifying the initial signal (Garcia de Herreros and Baulida, 2012). On the contrary, Snail1 also represses its own synthesis, either by direct binding to SNAIL promoter or by blocking the activity of Egr-1, a Snail1-gene transcriptional activator (Grotegut et al., 2006; Peiro et al., 2006). A theoretical analysis of these circuits has suggested that the EMT process is bi-stable, and an intermediate epithelial-mesenchymal hybrid phenotype also presents stability (Lu et al., 2014). Another study using human breast epithelial MCF10A cells treated with TGF-β also describe the existence of this intermediate phenotype that, contrarily to the full mesenchymal state, is reversible upon elimination of the cytokine (Zhang et al., 2014). The existence of this partial EMT is consistent with observations in carcinomas where co-expression of epithelial and mesenchymal markers has been detected in the same cells. However, in most tumors this intermediate phenotype looks much more epithelial than mesenchymal and maintains most of the epithelial structures, which suggests that the balance is biased toward the epithelium linage.

## E-cadherin protein protects from EMT

**Figure 2:**
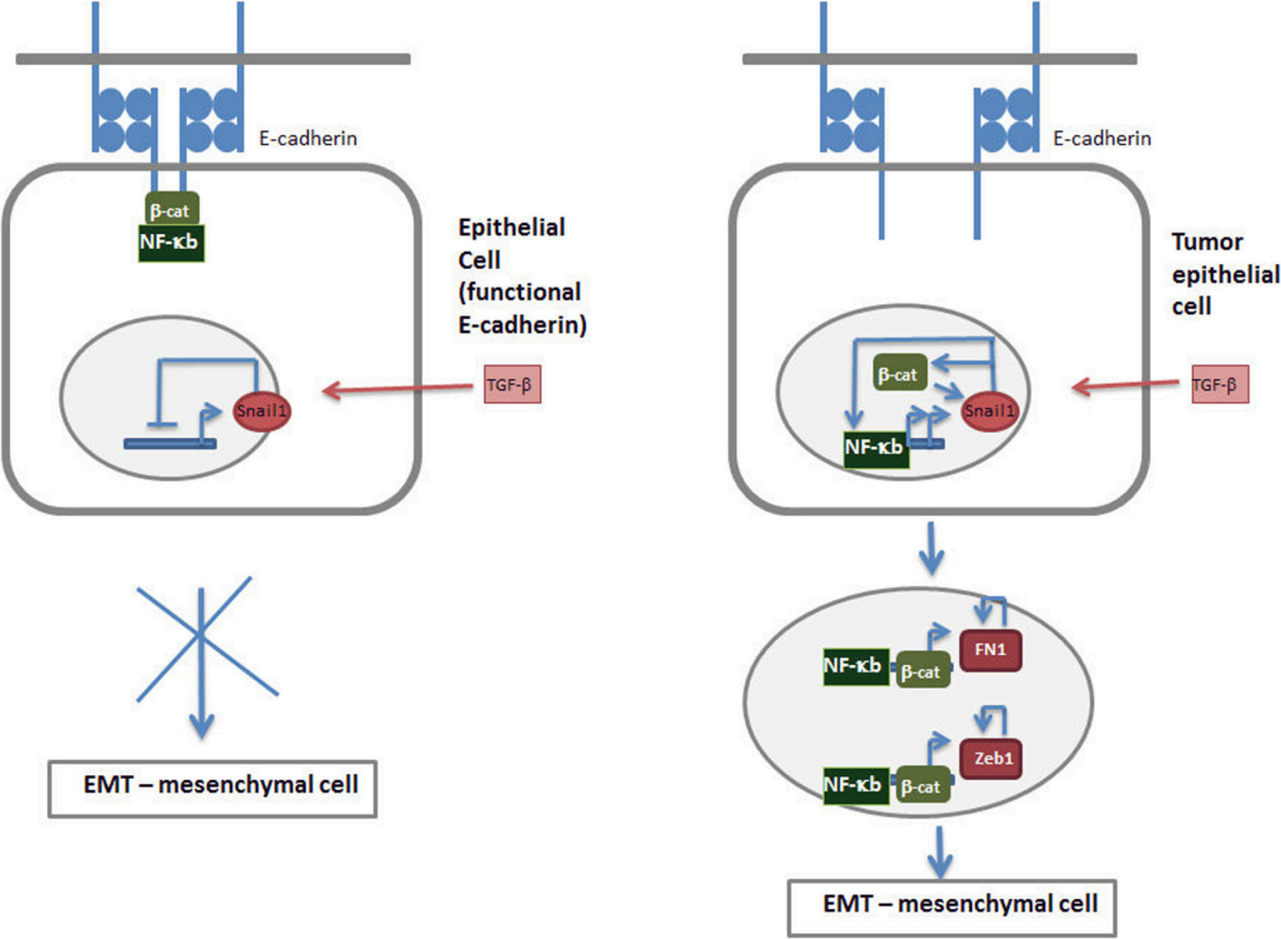
**Tumor cells are more receptive to signals inducing EMT**. According to our model, an epithelial cell with functional E-cadherin and stable adherens junctions is not sensitive to extracellular signals inducing Snail1 and EMT such as TGF-β **(left)**. Although these cytokines activate Snail1 expression, due to the high stability of E-cadherin protein the down-regulation in E-cadherin mRNA is not translated in changes in the protein. Moreover, stable junctional complexes retain β-catenin and NF-κB restrained their traffic to the nucleus and transcriptional activity. In these conditions Snail1 expression is self-inhibited and the EMT process is not initiated. Tumor epithelial cells contain less static adherens junctions due to post-translational modifications of E-cadherin or associated proteins, such as β-catenin; consequently the stability of E-cadherin protein is lower. In these conditions Snail1 increases do cause a significant down-modulation in E-cadherin protein and facilitates the traffic of NF-κB ad β-catenin to the nucleus and the activation of their transcriptional activity. These two factors further stimulate Snail1 expression, either by directly stimulating Snail gene transcription (NF-κB) or increasing protein stability (NF-κB, β-catenin), therefore amplifying the initial signal. Moreover, they also up-regulate the transcription of other mesenchymal markers such as Zeb1 or fibronectin (FN1), triggering a self-stimulatory circuit described in Figure 1 and supporting the EMT.

The most relevant gene product for the maintenance of the epithelial phenotype is E-cadherin. As it is well known, E-cadherin is required for the formation of adherens junction; however its role is not limited to this since it binds and restrains the transcriptional activity of β-catenin (Valenta et al., 2012) that, as commented above, is required for the expression of EMT-TFs. A similar negative action of E-cadherin has also been reported on NF-κb activity (Kuphal et al., 2004; Solanas et al., 2008). Although in this case the biochemical basis is not well characterized it relies in the interaction of NF-kB with the adherens junction complex (Deng et al., 2002; Solanas et al., 2008). This E-cadherin role limiting the transcriptional role of β–catenin, NF-κB and perhaps Ets-2 is essential for the control of EMT and the activity of EMT-TFs. Accordingly, E-cadherin ectopic expression prevents the induction of mesenchymal genes in several models of EMT (Ohkubo and Ozawa, 2003; Solanas et al., 2008). Although it is not sufficient by itself, since only very occasionally E-cadherin down-regulation induces mesenchymal markers, the decrease in the levels or function of this protein is absolutely necessary for a full EMT. As we present in our model, shown in Figure 2, cells presenting static adherens junctions are not receptive for signals promoting an EMT. Although Snail1 is induced and eventually down-regulate E-cadherin gene transcription, since E-cadherin protein when present in the adherens junction is protected from degradation (Gumbiner, 2005), this Snail1-induced mRNA decrease will not be translated in a down-regulation in E-cadherin protein. In addition, the stable junctional complexes retain β-catenin and NF-kB and restrain their traffic to the nucleus and their transcriptional activity on mesenchymal genes (Kuphal et al., 2004; Solanas et al., 2008). Therefore, E-cadherin acts as safeguard of the epithelial phenotype preventing transient signals to be amplified and promote undesired EMT. Thus, this transition will require additional signals that decrease adherens junction stability and make cells receptive to EMT-TFs action.

## Tumor cells are prone to EMT

Accordingly, it is expected that epithelial cancer cells will be much more susceptible to EMT than non-transformed epithelium (Figure 2). Total or partial loss of adherens junctions is a common alteration in neoplastic cells normally due to post-translational modifications of E-cadherin-associated proteins; for instance β-catenin tyrosine phosphorylation decreases its interaction with E-cadherin and causes adherens junction instability (Lilien and Balsamo, 2005). This modification is induced by ras or growth factor receptors, elements of a pathway commonly activated in many types of cancers. Moreover, other signaling pathways can weaken E-cadherin with p-120-catenin and favors E-cadherin degradation (Kourtidis et al., 2013). Therefore, transformed epithelial cells can be considered as “primed” epithelial cells capable to respond to cytokines such as TGF-β initiating an EMT.

Cell culture studies add a further level of instability to the phenotype. Tumor cell lines are normally cultured in the presence of high serum concentration, a condition that stimulates growth factor receptors. In these conditions many cells do not form compact colonies, at least at low confluence what makes adherens junctional complexes more unstable. Actually, Snail1-induced EMT is much more rapid and complete when cells are cultured at low confluence since the effect of this TF is much more rapidly translated in changes in E-cadherin protein levels. Even without additional signals, the phenotype of some tumor cells within the intermediate state is very much dependent on the confluence showing an epithelial morphology at high density and a more mesenchymal one at low confluence (Conacci-Sorrell et al., 2003).

Therefore, it is likely that many of the factors reportedly involved in the control of EMT have only an effect restricted to specific tumor cells presenting a partially inactivated E-cadherin and likely accumulating other alterations. Due to their intrinsic genomic instability, tumor cells might have alterations in genes controlling the EMT-TFs; for instance, a mutation in one of the ubiquitin ligases controlling Snail1 might enhance Snail1 up-regulation by factors activating Snail1 transcription. Once Snail1 have reached a threshold, the EMT process would be amplified by the self-stimulatory circuits and the loss of safeguard mechanisms. These alterations can affect to the many gene products acting on the different levels of control of EMT; thus on EMT-TFs transcription, mRNA stability or protein half-life. Thus, tumor cells would not just present genetic but also phenotypic instability, an alteration exacerbated in culture conditions.

Another interesting point is the discrepancy between the results obtained *in vitro*, in cell lines, showing that EMT is induced by many different conditions and factors, and *in vivo*, in human tumors, where few cells are detected that have undergone or are in the process of undergoing an EMT (Thompson et al., 2005). It is possible that in human tumors the cellular conditions (cell to cell contacts, composition of tumor stroma) do not enable a full EMT and most cells only respond partially to the same conditions than *in vitro* promote a very extensive transition. In any case, this partial EMT obtained *in vivo* might result in the acquisition of some mesenchymal markers and increased invasion maintaining most of epithelial features. It is expected that this EMT is more easy to be observed in the tumor border, where epithelial cell-to-cell interactions are more relaxed due to fewer physical constrains and also to the existence of extracellular cues provided by stromal cells.

Therefore, although nothing can be dismissed in cancer, it is likely that many of the described regulators of EMT present a very limited relevance *in vivo*. The physiological implication of the many proteins reported to be involved in EMT requires further investigation, not limited to a specific cell line in culture but to animal models or at least to a panel of cell lines. This would allow unveiling the most relevant gene products involved in the control of this essential cellular conversion.

### Conflict of interest statement

The author declares that the research was conducted in the absence of any commercial or financial relationships that could be construed as a potential conflict of interest.
